# Ionic conductivity of melt-frozen LiBH_4_ films†

**DOI:** 10.1039/c9ra06821j

**Published:** 2019-11-27

**Authors:** J. Trück, E. Hadjixenophontos, Yug Joshi, G. Richter, P. Stender, G. Schmitz

**Affiliations:** University of Stuttgart, Department of Materials Science, Chair of Materials Physics Heisenbergstrasse 3 70569 Stuttgart Germany Guido.Schmitz@mp.imw.uni-stuttgart.de; Institute of Engineering Thermodynamics, German Aerospace Centre (DLR) Pfaffenwaldring 38-40 70569 Stuttgart Germany; MPI for Intelligent Systems Stuttgart Heisenbergstrasse 3 70569 Stuttgart Germany

## Abstract

The fast Li conductivity of LiBH_4_ envisages its use in all-solid-state batteries. Powders are commonly applied. But here, we study the formation of dense micrometer films by melting, spinning and subsequent solidifying. Characterized by electron microscopy, and spectroscopy (EDX/XPS/impedance), a reversible phase transformation is confirmed as well as a maximum conductivity of 10^3^ S cm^−1^.

## Abstract 2

LiBH_4_ melt-frozen film as solid state electrolyte.

## Graphical abstract

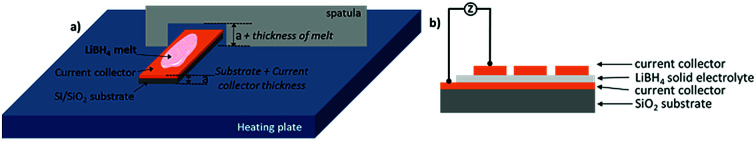

Li ion batteries exhibit a high gravimetric and volumetric capacity as well as a high power density.^1^ To overcome the drawbacks of liquid electrolytes such as safety concerns, leakage and limitations for miniaturization, solid state batteries are a suggested alternative.^2^ In this context, complex hydrides have attracted much interest as candidates for solid state electrolytes. Lithium borohydride (LiBH_4_) is a promising representative of this group.^3^

Conventionally, LiBH_4_ is applied as reducing agent, while recent research focuses on its energy functions such as solid state hydrogen storage, electrochemical Li storage and fast Li-ionic conductivity. When Matsuo *et al.* discovered the fast ionic conductivity of LiBH_4_ in 2007,^4^ the research on its potential application as electrolyte was initiated.

The electrical properties of LiBH_4_ are related to its crystal structure. The low-temperature (LT) orthorhombic phase (space group *Pnma*) causes Li ionic conductivities in the range of 10^−8^ to 10^−6^ S cm^−1^. At 110 °C, the transformation to the hexagonal high-temperature (HT) phase (space group *P*6_3_*mc*) induces a discontinuous jump in the conductivity^4^ to 10^3^ S cm^−1^, which is in the same range as offered by liquid electrolytes. The fast Li ionic conduction in LiBH_4_ was theoretically justified.^5^ It was experimentally studied with pelletized samples (≈2 mm thickness) made of pressed LiBH_4_ powder^4^ and with nanoconfined LiBH_4_ in pores of ordered silica scaffolds.^6^ Due to its hygroscopicity, handling of LiBH_4_ is restricted to protected atmosphere. In contact with steam and oxygen, the compound reacts spontaneously to hydroxides and oxides and a large amount of hydrogen is released.^7,8^

Xiong *et al.* prepared LiBH_4_ films by pulsed laser deposition under hydrogen atmosphere from a LiB target to investigate the hydrolysis of the material.^9^ However, the electrochemical characterization of LiBH_4_ films has not yet been reported. Thin films are advantageous for studying diffusion coefficients as well as the interfaces with different electrodes. Thin films may also open a way to improve the intimate contact between solid electrolyte and electrode. Currently, LiPON (lithium phosphorous oxynitride) is the most often applied thin film electrolyte, which shows conductivities of 10^−6^ S cm^−1^ at RT and could be prepared with a thickness^10^ down to 12 nm.

The purpose of the present study is preparing thin films of LiBH_4_ and characterizing their microstructure and ionic conductivity. Melting of LiBH_4_ powder and solidifying in layer geometry turns out to be a promising process. The method is enhanced by spin coating of the molten LiBH_4_ aiming for thinner films. Electron microscopy (SEM) is used for measuring the thickness of the films. Photoelectron and energy dispersive X-ray spectroscopy (XPS, EDX) are used for chemical analysis while impedance spectroscopy (EIS) characterizes the ionic conductivity.

Substrates were cut from silicon (Si) wafers with a thickness of (675 ± 15) μm and an orientation (111), which were previously oxidized by annealing for 5 hours at 1200 °C under air. On top, 50 nm of different current collectors, copper (Cu), platinum (Pt) or tantalum (Ta), were deposited by ion beam sputtering (IBS) in a custom-built UHV chamber with a DC ion source (KF/F 40, Ion-Tech GmbH). The LiBH_4_ powder was purchased from Albemarle. Due to the sensitivity of LiBH_4_ to oxygen and moisture, all experiments were conducted in protecting Ar atmosphere in a glove box (contents of oxygen and water ≤ 0.5 ppm) and the transfer to other instruments was protected when possible. For the melting process, the substrate with the powder was placed on a heating plate set to 290 °C (inside the glove box) and afterwards the LiBH_4_ melt was flattened with a spatula. A notch in the spatula served as mechanical spacer to control the thickness of the film. Alternative spin coating was carried out on a KLM spin coater SCE (Schaefer) inside the glove box, with a custom-made sample holder that could be heated on a hot plate and offered sufficient heat capacity to keep the ion conductor liquid during the subsequent spinning.

Upon the LiBH_4_ layer, the upper current collector was deposited by IBS in a dotted electrode pattern to create multiple cells on a single sample wafer. The dots were of 1 mm diameter and the current collector thickness on top was varied between 50–150 nm. The samples were transported from the glove box to the IBS sputter chamber in an argon filled container.

Electrical impedance spectroscopy was carried out directly after the film preparation, inside the glove box with the potentiostat VSP-300 (BioLogic). Samples were electrically contacted by gold coated stainless steel tips (Bürklin: 11H5560). Conductivity was measured at temperatures between room temperature and 150 °C. The frequency range was 3 MHz to 1 Hz and voltage amplitude up to 2.5 V.

For microstructure imaging and EDX analysis, a DualBeam microscope (ThermoFischer Scios) was utilized for which protected transfer from the glove box was achieved with the vacuum shuttle LEICA EM VCT500. The XPS measurements were conducted with a Theta Probe ARXPS (ThermoFischer Scientific) for which mounting of the samples required a few seconds of atmosphere contact.

A sketch of the layer preparation is shown in Fig. 1(a) and the geometric layer setup for the impedance measurements in Fig. 1(b). The samples were heated inside a glove box to 290 °C to ensure melting but avoiding decomposition of the hydride. The thickness of multiple electrolyte films flattened with the spatula was measured by FIB cross-sections to an average thickness of (18.6 ± 6.5) μm. This shows that layers with a reasonably reproducible thickness can be prepared by this method. An exemplary cross-section is shown in Fig. 2(a), where the upper and lower Cu current collectors are also visible. Pores visible in the layer are attributed to hydrogen loss by the ion beam cutting, which is observed also in other even more stable hydrides.^11^ On the cross-section, EDX analyses were carried out as depicted in Fig. 2(b). In both spectra similar amount of boron is detected. Spectrum 1, measured at the surface of the layer, reveals a more significant contribution of oxygen (O). This indicates a slight surface reaction, taking place during sample transfer from the glove box to the ion beam sputter chamber. This observation confirms the high sensitivity of the material to the atmosphere (also discussed in Fig. 4(a)). Oxygen compounds are the typical products obtained when the borohydride comes into contact with air.^7,8^ Additionally, Si from the substrate is visible in the second spectrum due to the tilt of the sample for cross section – EDX analysis. Chlorine also visible in both spectra is an impurity present in the purchased LiBH_4_ powder.

**Fig. 1:**
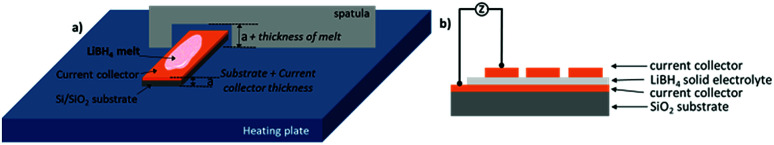
(a) Sketch of the preparation of LiBH_4_ layer on the heating plate by flattening the melt with a spatula containing a cut with a depth of 0.7 mm. (b) Final sample design. The current collectors were prepared by ion beam sputter-deposition. Different current collector materials are compared: Cu, Pt or Ta.

**Fig. 2:**
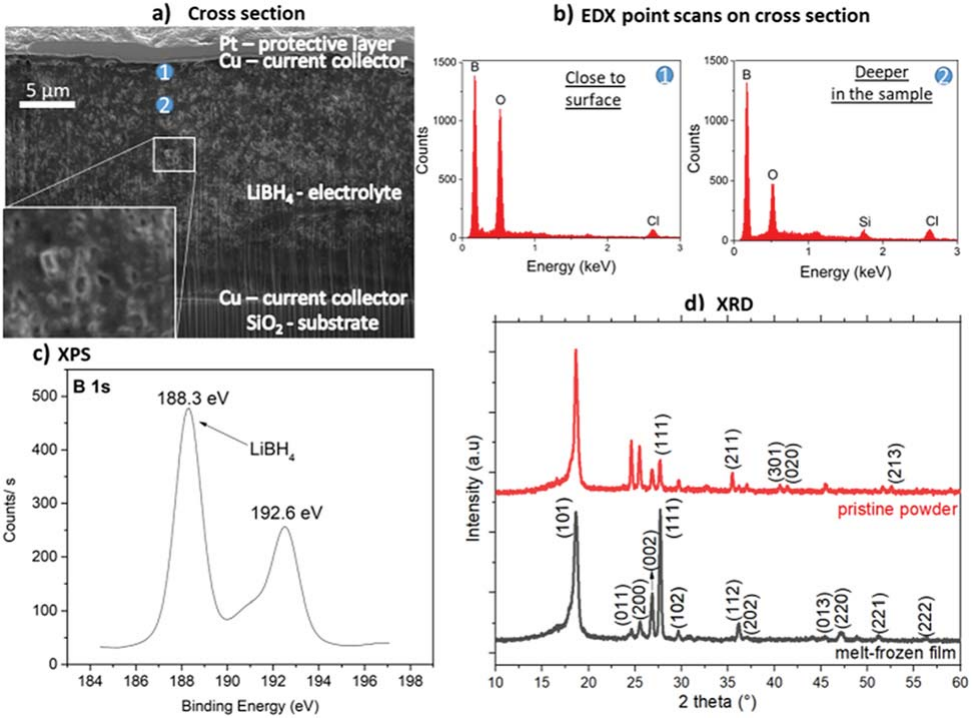
Investigations of a LiBH_4_ layer prepared by melt-freezing of LiBH_4_ powder on a SiO_2_ substrate with ion beam sputter-deposited Cu 50 nm as current collector: (a) SEM image (5 kV, 0.8 nA) of a FIB cross-section with the positions of the EDX analyses marked. (b) EDX spectra of the two positions marked in a: 1 – closer to the surface and 2 – deeper in the layer volume. (c) XPS measurement showing the B 1s peak at 188.3 eV confirming the major presence of LiBH_4_.^12,13^ (d) XRD spectrum of the pristine LiBH_4_ powder used and the melt-frozen film. All major (indexed) reflexions are understood by the crystalline LT phase of LiBH_4_.

Fig. 2(c) shows the XPS characterization of the produced LiBH_4_ layer. Despite the oxide also visible in the EDX analyses, the peak at 188.3 eV corresponds to LiBH_4_,^12,13^ clearly confirming that the desired compound is present to a major fraction after melting and solidification. The peak at 192.6 eV is attributed to LiB_4_O_7_ which probably represents the oxidation product formed during the transport of the sample to the XPS. The effect this oxidation layer has on hydrogen desorption was previously reported,^12,15,16^ however its effect on the ionic conductivity in battery applications has not yet been considered. XRD characterization of the melt-frozen samples was performed to confirm their crystallinity after melting and cooling. The XRD is shown in Fig. 2(d) revealing the crystalline nature of the sample. The peaks identify the LT phase of LiBH_4_. For comparison the XRD of the pristine powder used for the preparation is given and additional EDX of the powder is shown in the ESI.†

Nyquist plots of the impedance spectroscopy are presented in Fig. 3(a). The LT phase response appears with a complete semi-circle, while the HT phase does not start at the origin, an observation also visible in the work of Matsuo *et al.*^4^ It can be attributed to the high frequency limit of the potentiostat.

**Fig. 3:**
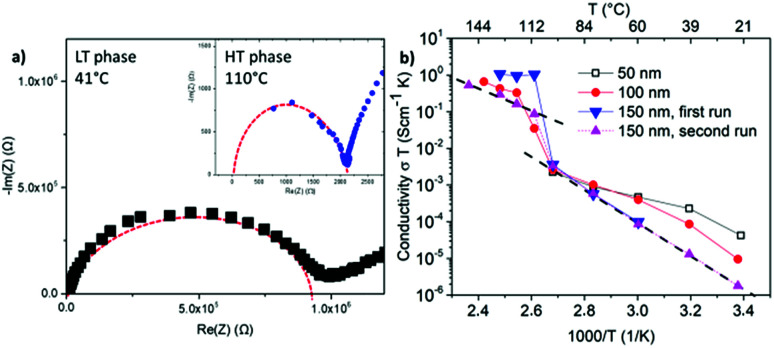
(a) Nyquist plots of the LT and the HT phase of LiBH_4_ layers prepared by flattening the melt with a spatula and contacted with a Cu current collector. Model curves of the replacement circuit in red dashed. (b) Arrhenius plots of ≈18 μm LiBH_4_ layers prepared by flattening the melt with a spatula on a 50 nm Cu current collector as bottom contact but different thicknesses of the upper Cu current collector as labelled.

The calculated conductivities of three independent samples are provided in Fig. 3(b). In these measurements, the thickness of the upper Cu current collector was stepwise increased: 50 nm, 100 nm and 150 nm. The impedance spectroscopy of the sample with the 50 nm thin upper current collector, shown in black squares, could only be operated in the LT phase. At higher temperature no impedance could be measured. Increasing the thickness of the upper current collector to 100 or 150 nm, enabled reliable measurements of both the LT and the HT phases. This suggests damaging of the upper current collector layer or its interface towards the electrolyte during the phase transition. On the other hand, repetition of the heating cycle of the sample with an upper current collector of 150 nm in thickness (see triangles) demonstrates the reproducibility of the measurements and so the microstructure stability during phase transition. For later measurements the thickness of the upper current collector was chosen to be 150 nm.

All resistivity measurements show the discontinuous jump of two to three orders of magnitude in conductivity at around 110 °C indicating the phase transition from orthorhombic to hexagonal. Below and above this discontinuity, the temperature dependence shows the expected Arrhenius behavior. In the LT phase, the conductivity ranges from 10^−7^ to 10^−8^ S cm^−1^ at room temperature to 10^−5^ to 10^−6^ S cm^−1^ before phase transition. The HT phase reaches values of ≈10^−3^ S cm^−1^. These observations are in accordance with the measurements of Matsuo *et al.*^4^ at compressed powders, which demonstrated a maximum conductivity of 10^−3^ S cm^−1^ at the HT phase. They confirm the presence of LiBH_4_ in the melt-frozen layers and demonstrate the fast ionic conductivity in the HT phase which is comparable to the conductivities of liquid electrolytes. The activation energies for the conductivity in the LT and HT phase amount to (0.89 ± 0.1) eV and (0.60 ± 0.1) eV, respectively. They are slightly higher than the values of 0.69 eV for the LT and 0.53 eV for the HT phase reported for the powder.^4^ The difference is probably due to the more compact material in the molten films with less pores and grain boundaries in comparison to the compressed powders.

Since these layers could be applied as thin film membranes in solid state batteries, improvement of the process could result in thinner layers, which was attempted by spin coating of the LiBH_4_ melt. Indeed, the method can decrease the layer thickness. The following values were found for coating on Cu: (5.3 ± 0.8) μm for 30 rps, (2.3 ± 0.3) μm for 60 rps, (1.5 ± 0.2) μm for 90 rps, (1.3 ± 0.2) μm for 120 rps. Fig. 4(a) shows a cross-section of a sample coated with 30 rps with an overlaid EDX scan. Similar to the EDX spectra shown before, the O content is significantly elevated near the surface of the LiBH_4_ layer. A sharp contrast visible in the secondary electron image between the contaminated surface layer with increased O content and the LiBH_4_ volume underneath is partly marked by a dashed line. This indicates a clear reaction front between the surface oxide and the remaining LiBH_4_ volume. Platinum (Pt) and silicon (Si) are detected because they are the current collector and the substrate, respectively in this case. (The apparent decrease in the Boron content is a consequence of different sample tilt during the EDX measurement.) The sharp contrast in oxygen intensity as well as the contrast in the electron image strongly indicates that in this case indeed a “second” surface phase has formed. In the case of the thinner spin-coated films, this layer also affects the impedances spectra as it will be further discussed hereafter.

**Fig. 4:**
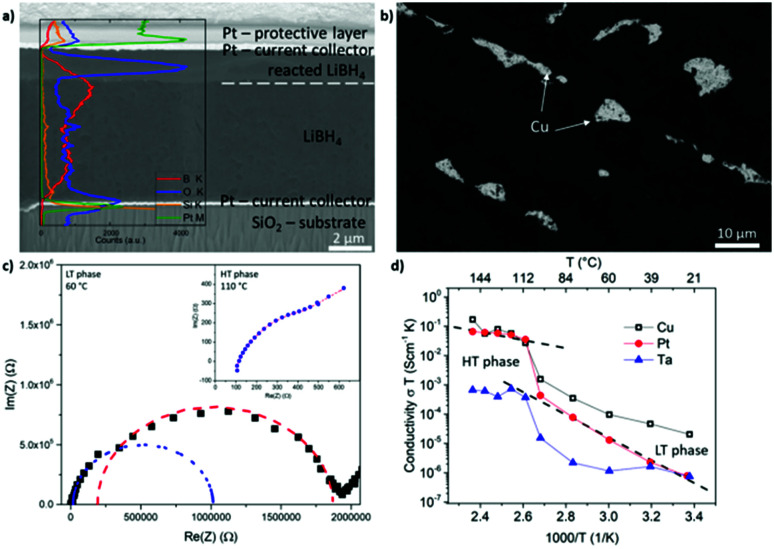
(a) SEM picture (5 kV, 1.6 nA) of a FIB cross-section trough a LiBH_4_ layer spin coated with 30 rps on a Pt current collector. Overlaid EDX line scans discover an increased O content in the surface layer of the LiBH_4_. (b) Top view on a LiBH_4_ layer, spin coated with 90 rps on Cu a current collector imaged with backscattered electrons (5 kV, 0.8 nA). The bright spots are uncoated Cu areas. (c) Nyquist plot of the LT and the HT phase of a LiBH_4_ layer prepared by spin coating with 30 rps on a Pt current collector. (d) Arrhenius plots of LiBH_4_ layer prepared by spin coating with 30 rps with different current collectors.

As a drawback, the spin-coated LiBH_4_ layers do not completely cover the current collector, as it is seen in the top view of the backscattered electrons image in Fig. 4(b). Furthermore, the fraction of uncoated substrate area increases with increasing rotation speed, discovering this way a decisive disadvantage of the spin coating method. Since the upper current collector is sputter-deposited upon the LiBH_4_ layer, holes in the ion conductor layer lead to short circuits. Due to these short circuits, only layers coated with 30 rps could be measured by impedance spectroscopy, which formed fewer short circuits. In the case of the Cu current collector only 4.1% of the contact dots examined did not reveal a short circuit. In order to check, whether the fraction of coating can be improved by better wetting of different metals, Pt and Ta were also tested as current collectors (upper and lower). Their higher melting temperature and therefore higher surface tension promise better wetting. This however did not improve the problem significantly. In the case of Pt, 5.3% of the dots examined on different samples did not reveal a short circuit. In the case of Ta, the quote was even worse, only 1.6%.

Representative Nyquist diagrams of the LT and the HT phase are shown in Fig. 4(c). The measurement of the LT phase differs to that of the layers flattened with the spatula (compare Fig. 3(b)). This time, two semi-circles are visible showing that an additional resistance is present. By using an equivalent circuit with 2 resistances in series the fitting and interpretation of these two semi-circles is possible. The corresponding fit is shown in blue for the first semi-circle and in red for the second semi-circle. Expecting the lower conductivity for the thicker LiBH_4_ film, we can conclude that the ionic conductivity of the main LiBH_4_ is represented by the blue semicircle at higher frequency, while the second red one is attributed to the surface layer observed in Fig. 4(a). Naturally, in the case of the spin coated samples where the total thickness of the layer is smaller, the oxide surface layer plays a more important role. At the HT phase, only one semi-circle is visible but it is overlapped partly by the interface response. Also an inductive contribution becomes visible in comparison to the low absolute resistance. Thus, the accuracy of the HT conductivity of the spin-coated films is limited.

Arrhenius plots of the conductivity measured for the different current collectors are provided in Fig. 4(d). The measurements with Cu and Pt show the characteristic shape and in the HT phase a conductivity of 10^−3^ S cm^−1^ is reached. Nevertheless, the conductivity is slightly lower than observed for the thicker films of the spatula-flattening method. This might be attributed to the discussed surface reaction during the sample transfer from the glove box to the sputter chamber. Since the spin coated layers are one order of magnitude thinner, a surface reaction of similar reaction depth affects the conductivity of the spin coated layers more significantly. In the case of Ta, the conductivity stays below 10^−5^ S cm^−1^ in the entire temperature range. From literature it is found that Ta surfaces develop a passivating tantalum pentoxide film^14^ with which, most likely, the LiBH_4_ reacted in this case, resulting in the lower conductivity and the many short circuits of these samples. This indicates Ta as a bad candidate for a current collector in contact to LiBH_4_. The ESI† presents an additional comparison to pressed powder pellets showing a very similar temperature dependence of the conductivity.

LiBH_4_ has recently shown ionic conductivity comparable to the current liquid electrolytes used in Li-ion batteries. While powders are in the focus of interest to produce all-solid-state full cells, thin films can be a favourite tool to investigating the Li transport in these materials and promise more reliable dense structure at lower thicknesses. Two methods for preparing LiBH_4_ layers for solid-state electrolyte testing from LiBH_4_ melt with reproducible layer thickness were established during this work. Flattening the melt with a spatula leads to a thickness of 18.6 ± 6.5 μm. Additional spin-coating reduces the thicknesses to 5.3 ± 0.8 μm and even to 1.3 ± 0.2 μm depending on the rotation speed. However, frequent short circuits in the EIS measurements of the spin coated layers were induced because the LiBH_4_ layer did not wet the bottom metallization completely. Different current collectors such as Cu, Pt and Ta were tested as substrate in pursuit of improvement. Nonetheless a high amount of short circuits was always visible. By contrast, the layers flattened with a spatula revealed a high reversibility and reliability in electrical measurement.

The ionic conductivity of the layers flattened with a spatula reached in the HT phase ≈10^−3^ S cm^−1^, a value comparable to liquid electrolytes. Nevertheless, high temperatures of 110 °C are necessary for the transformation into the conductive HT phase, which is a rather high temperature for applications. The spin-coated layers showed a reduced conductivity due to a surface reaction that takes place during the transfer between tools. The oxidation product layer is clearly visible in the SEM cross section and its additional resistance is observed with a second semi-circle in the EIS measurements. The high amount of oxygen in the contamination layer, is visible in the EDX and oxides are also detected by XPS measurements. Despite the presence of oxygen, XPS measurement and the characteristic temperature dependence of the conductivity confirm that the produced layers consist dominantly of LiBH_4_. This work establishes an auspicious method for creating solid state thin film batteries with this hydride.

## Conflicts of interest

There are no conflicts to declare.

## Supplementary Material

RA-009-C9RA06821J-s001
