# Supplementary material for: Ionic conductivity of melt-frozen LiBH4 films
Source: RSC Adv. 2019 Nov 27;9(66):38855–9. doi: 10.1039/c9ra06821j (PMC9075933; doi:10.1039/c9ra06821j)
Supplement: RA-009-C9RA06821J-s001 [file RA-009-C9RA06821J-s001.pdf]

Electronic Supplementary Material (ESI) for RSC Advances.
This journal is © The Royal Society of Chemistry 2019

Supplementary information

The commercial $LiBH_4$ powder used as starting material for the produced films was characterized by XRD and EDX as shown in Figure s1. The XRD pattern in Figure a) is in good accordance with patterns of the LT phase in the literature (Aoki et al. 2005; Oguchi et al. 2009). Si is visible in the XRD since Si wafer was used as a substrate for transferring the powder in the XRD. Mylar foil for keeping the XRD sample under Ar atmosphere was used and is visible with the amorphous broad peak at low 2 theta angles. In the EDX measurement in Figure b), the B peak is the only indicator for the complex hydride, since Li and H can't be detected by EDX. This also means that no quantitative elemental analysis of this compound is possible by EDX. Carbon (C) is often observed in SEM/ EDX as an electron beam-induced surface contamination. The presence of chlorine (Cl) is attributed to impurities of the purchased powder. However, it is not considered to affect the properties of the compound strongly, since the XRD pattern shows clearly the expected structure. The oxygen (O) peak stems from an oxidation reaction of the powder during its transfer from the glove box to the SCIOS dual beam instrument with the shuttle. Hence, the shuttle could not prevent the contamination of the sample from the atmosphere completely. This shows the strong sensitivity of the sample to air.

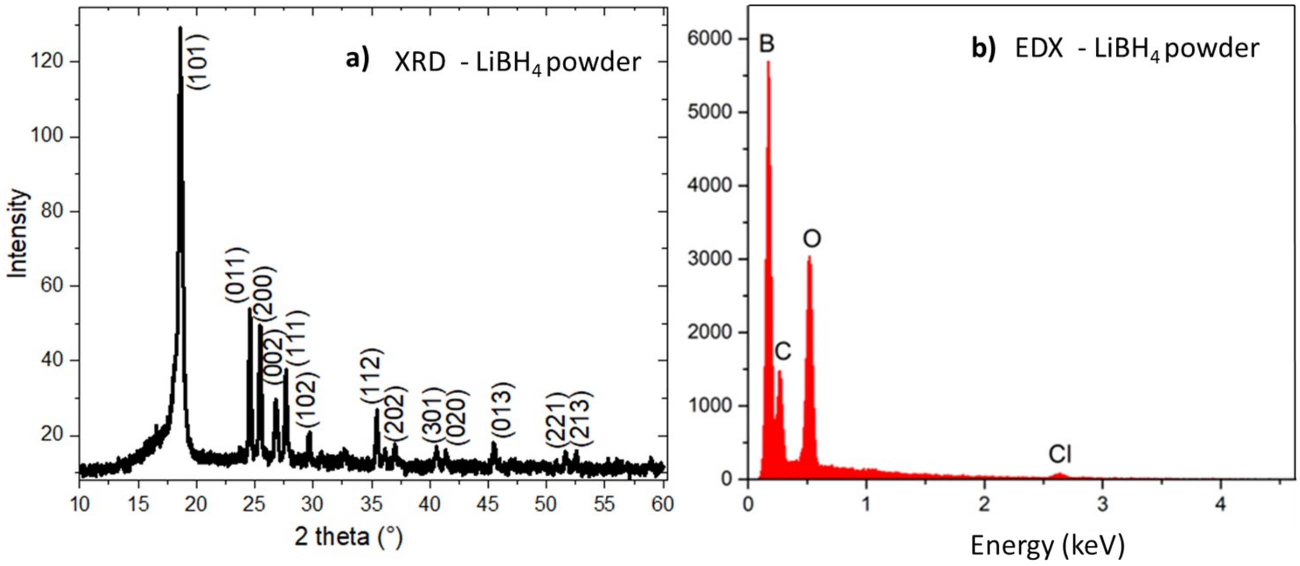

Figure s1: XRD and EDX of the pristine powder used for the preparation of the samples. The powder shows the low temperature phase of $LiBH_4$.

Supplementary comparison of pressed powder samples to the melt-frozen film samples was done. Figure s2 shows a schematic of the pellets pressed within Li electrodes and stainless steel. Impedance measurements were performed in the same conditions as the film samples. The same powder as used for the molten-frozen films was pressed with 127MPa to 382MPa. The different pressures used give rise to different porosity of the electrolyte. The cells were assembled in an Ar glove box using Swagelok cells. In comparison, the ionic conductivities of the pressed powders and of the melt-frozen films are shown to be in the same range. Furthermore, the change from the LT to HT phase of $LiBH_4$ is clearly visible. The stability of the samples is demonstrated here with the filled symbols representing the 1st heating ramp while the empty symbols representing the 10th.

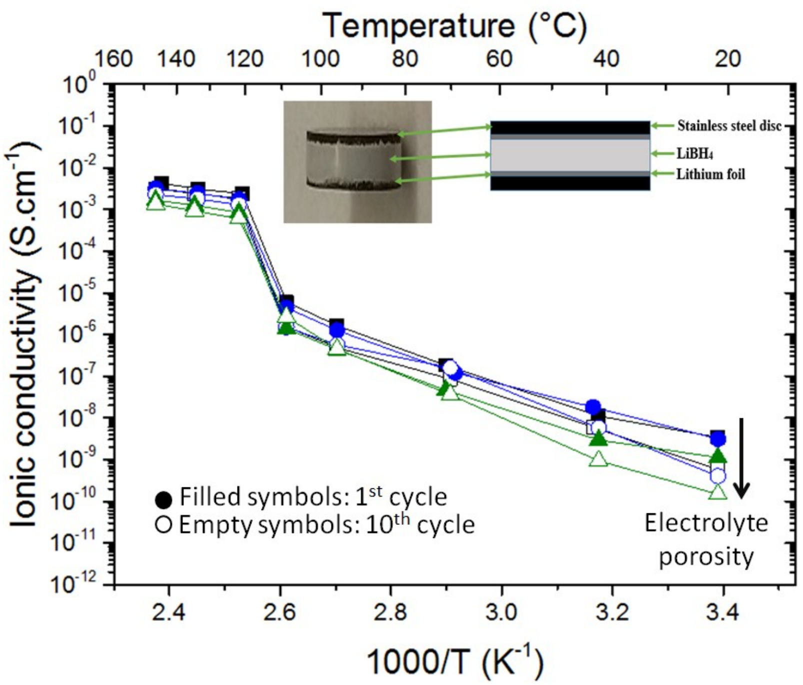

Figure s2: Ionic conductivity of pressed $LiBH_4$ powder within Lithium electrodes. The filled symbols represent the 1st measurement and the empty symbols represent the 10th measurement. Additional schematic of the tested powder is shown with the pressed pellet visible between the discs.

Publication bibliography

Aoki, M.; Miwa, K.; Noritake, T.; Kitahara, G.; Nakamori, Y.; Orimo, S.; Towata, S. (2005): Destabilization of LiBH4 by mixing with LiNH2. In *Appl. Phys. A* 80 (7), pp. 1409–1412. DOI: 10.1007/s00339-004-3194-9.

Oguchi, H.; Matsuo, M.; Hummelshøj, J. S.; Vegge, T.; Nørskov, J. K.; Sato, T. et al. (2009): Experimental and computational studies on structural transitions in the LiBH4–LiI pseudobinary system. In *Appl. Phys. Lett.* 94 (14), p. 141912. DOI: 10.1063/1.3117227.
